# Hepatitis B, hepatitis C, and mortality among HIV-positive individuals

**DOI:** 10.1097/QAD.0000000000001646

**Published:** 2017-11-09

**Authors:** Alicia C. Thornton, Sophie Jose, Sanjay Bhagani, David Chadwick, David Dunn, Richard Gilson, Janice Main, Mark Nelson, Alison Rodger, Chris Taylor, Elaney Youssef, Clifford Leen, Mark Gompels, Stephen Kegg, Achim Schwenk, Caroline Sabin

**Affiliations:** Research Department of Infection and Population Health, Royal Free Campus, UCL, London, UK.

**Keywords:** cohort study, coinfection, hepatitis B, hepatitis C, mortality

## Abstract

**Objectives:**

To compare rates of all-cause, liver-related, and AIDS-related mortality among individuals who are HIV-monoinfected with those coinfected with HIV and hepatitis B (HBV) and/or hepatitis C (HCV) viruses.

**Design:**

An ongoing observational cohort study collating routinely collected clinical data on HIV-positive individuals attending for care at HIV treatment centres throughout the United Kingdom.

**Methods:**

Individuals were included if they had been seen for care from 2004 onwards and had tested for HBV and HCV. Crude mortality rates (all cause, liver related, and AIDS related) were calculated among HIV-monoinfected individuals and those coinfected with HIV, HBV, and/or HCV. Poisson regression was used to adjust for confounding factors, identify independent predictors of mortality, and estimate the impact of hepatitis coinfection on mortality in this cohort.

**Results:**

Among 25 486 HIV-positive individuals, with a median follow-up 4.5 years, HBV coinfection was significantly associated with increased all-cause and liver-related mortality in multivariable analyses: adjusted rate ratios (ARR) [95% confidence intervals (95% CI)] were 1.60 (1.28–2.00) and 10.42 (5.78–18.80), respectively. HCV coinfection was significantly associated with increased all-cause (ARR 1.43, 95% CI 1.15–1.76) and liver-related mortality (ARR 6.20, 95% CI 3.31–11.60). Neither HBV nor HCV coinfection were associated with increased AIDS-related mortality: ARRs (95% CI) 1.07 (0.63–1.83) and 0.40 (0.20–0.81), respectively.

**Conclusion:**

The increased rate of all-cause and liver-related mortality among hepatitis-coinfected individuals in this HIV-positive cohort highlights the need for primary prevention and access to effective hepatitis treatment for HIV-positive individuals.

## Introduction

Since the introduction of combination antiretroviral therapy (cART) for the treatment of HIV infection, rates of AIDS-related morbidity and mortality have fallen. HIV-positive individuals who are diagnosed promptly and have good access to cART are now expected to live a near normal lifespan. When HIV-positive individuals do die, they are less likely to die from AIDS-related causes than in the pre or early cART era. However, the proportion of people who die from liver-related causes has increased, with these deaths largely being related to coinfection with either hepatitis B virus (HBV) and/or hepatitis C virus (HCV) [1–5].

HIV-positive individuals coinfected with HBV and/or HCV have higher rates of progression to chronic hepatitis infection [6–8] and faster progression to fibrosis, cirrhosis, hepatocellular carcinoma, and end-stage liver disease [9–12] than individuals with hepatitis infection alone. Meta-analyses investigating the impact of HBV and/or HCV coinfection on mortality among HIV-positive individuals have shown that, overall, coinfection is associated with increased mortality compared with HIV alone [13,14]. However, findings from individual studies are inconsistent, possibly because of variations in underlying mortality rates in the cohorts studied as well as differences in treatment and management strategies for both HIV and hepatitis.

In a number of studies the association between HIV/HCV coinfection and increased mortality is lost after adjusting for HIV infection-related parameters (such as use of cART and CD4^+^ cell count) [15–18]. This could suggest that the observed increase in mortality risk is because coinfected individuals are less likely to initiate cART and/or experience a good immunological response to treatment. Similarly, the association between HIV/HBV coinfection and increased mortality is often found to be nonsignificant in multivariable models with similar mechanisms suggested [19,20]. As most studies that have examined mortality among coinfected individuals have reported on all-cause mortality, it is not possible to examine whether the apparent increase in mortality in coinfected persons is because of liver disease among coinfected individuals or because of other causes.

The aims of this study were to compare all-cause, liver-related, and AIDS-related mortality rates in coinfected individuals and HIV-monoinfected individuals in a large HIV-positive cohort in the United Kingdom.

## Methods

### Data collection and inclusion criteria

The UK Collaborative HIV Cohort (UK CHIC) study is an observational study collating data from some of the largest HIV clinics across the United Kingdom; the dataset used for the present analysis includes data from 11 clinics. Data are collected annually on demographics, HIV exposure group, HIV treatment, laboratory tests (CD4^+^ cell counts and HIV viral loads), and tests conducted to identify and classify hepatitis coinfection [21]. Clinics also report information on the date and cause of death, where recorded in the clinic database. Clinics are also invited to submit a standardized case report form (Coding Causing of Death in HIV form) for those individuals who are known to have died [22].

In September 2012, an expanded data collection process focussed on individuals from 11 UK CHIC centres who had evidence of coinfection with HBV [a positive hepatitis B surface antigen (HBsAg) test result] or HCV (a positive HCV antibody or positive HCV RNA test result). This involved detailed review of the clinical HIV notes of coinfected individuals seen for care from 2004 onwards. This process aimed to verify the hepatitis-coinfected individuals as well as collect detailed information on the treatment and outcomes of hepatitis infection among these individuals. As part of this process, additional information on the cause of death was collected for those individuals who were known to have died.

Individuals were included in this analysis if they had attended any one of the 11 centres where additional hepatitis data collection were conducted from 2004 onwards and had ever been tested for HBsAg and antibody to HCV (anti-HCV) or HCV RNA. Individuals were defined as being HBV infected from the date of their first positive HBsAg test and being HCV infected from the date of their first positive anti-HCV or HCV RNA test.

### Classifying cause of death

In addition to information on deaths received from clinics, for deaths prior to 2011, the UK CHIC dataset has been linked to data from the Office for National Statistics (ONS) using soundex codes, initials, date of birth, and sex. From 2011 onwards, the UK CHIC dataset was linked to mortality data from Public Health England (PHE). Where these linkages provided additional information on date and cause of death, the UK CHIC dataset was updated.

Causes of death were coded as liver related, AIDS related, or neither. Deaths were coded as liver related where there was clear evidence that disease in the liver had contributed to death. This included, for example, decompensated liver disease, hepatocellular carcinoma and liver failure, and cancers with liver metastases. Where a nonliver-related cause of death was recorded but there was additional mention of viral hepatitis, this was not coded as a liver-related death. Deaths were coded as AIDS related either where AIDS was stated as a cause of death or where the cause of death included conditions included on the Centres for Disease Control and Prevention list of AIDS-defining conditions [23]. Coding was done without knowledge of an individual's hepatitis status although there may have been reference to coinfection within the information recorded on cause of death. Causes of death received from each data source were coded separately and then combined. Where conflicting information was available, both sources were reviewed to ascertain the most likely cause of death.

### Statistical analysis

Individuals were followed from the latest of their first HBV or HCV test, entry into UK CHIC, or 1 January 2004. In some cases, individual deaths may be reported to a clinic a long time after the individual last attended the clinic. To prevent extended follow-up among these individuals, an additional 6 months of follow-up was added to all individuals. Subsequently, all deaths which occurred within 6 months of the last date that an individual was seen for care in UK CHIC were included in the analysis. Where the recorded date of death was more than 6 months after the last date which they were seen (15 individuals), follow-up was censored at 6 months after their last date of follow-up to prevent extended follow-up among those individuals who die. In addition, since different centres report data with varying cutoff dates, all data were censored at 31 December 2011.

Individuals were categorized at baseline as HIV monoinfected, HIV/HBV coinfected, HIV/HCV coinfected, or HIV/HBV/HCV triple infected. Individuals moved in one direction from HIV monoinfected to a coinfection or triple infection category if there was evidence of a positive test result during follow-up. The total number of person-years of follow-up was calculated for each coinfection category using the date at which an individual entered that coinfected category and their last date of follow-up within that category. Mortality rates were calculated as the total number of deaths divided by the total person-years of follow-up within each category.

Poisson regression was used to identify predictors of all-cause, liver-related, and AIDS-related mortality. Fixed covariates were ethnicity and HIV exposure group as reported by the clinics. Age, CD4^+^ cell count, HIV viral load, cART, and calendar year were updated every 3 months in the analysis. Hepatitis coinfection status was updated whenever a change occurred. Given the close relationship between HIV viral load and cART, these two variables were combined to form a composite covariate which focussed on whether or not viral load was detectable and whether or not an individual was receiving any cART: on cART and viral load less than 50 copies/ml; on cART and viral load 50–10 000 copies/ml; on cART and viral load more than 10 000 copies/ml; off cART and viral load less than 50 copies/ml; and off cART and viral load at least 50 copies/ml.

Final models were created using a backwards selection process to identify those factors that cofounded the effect of coinfection on mortality and to achieve a parsimonious model. Final models were adjusted for age, HIV exposure group, CD4^+^ cell count, HIV viral load and treatment, and year of follow-up. Although ethnicity was associated with mortality in univariable analysis, inclusion of ethnicity in the final models did not alter the effect of coinfection on mortality and therefore this was not included in the final models. Models of all-cause, liver-related, and AIDS-related mortality were adjusted for the same confounders to allow for comparison.

HIV-positive IDU have higher mortality rates than HIV-positive individuals who acquired their HIV infection through other routes [24–26]. In this cohort there is a very high prevalence of HCV coinfection among IDU (83%). Therefore, a sensitivity analysis was conducted where individuals who had acquired HIV through IDU were excluded.

## Results

### Mortality rates

A total of 25 486 individuals contributed 121 814 person-years of follow-up to the analysis. Median follow-up time was 4.6 years (interquartile range 2.0, 7.2 years) per person. Overall, 1065 individuals (4.2%) died during follow-up. Baseline characteristics of included individuals are shown in Table 1. The all-cause mortality rate was 8.7/1000 person-years [95% confidence interval (CI) 8.2–9.3]. A total of 95 individuals died of liver-related causes (a mortality rate of 0.8/1000 person-years, 95% CI 0.6–1.0) and 198 individuals died of AIDS-related causes (a mortality rate of 1.6/1000 person-years, 95% CI 1.4–1.9). All-cause and liver-related mortality was higher among coinfected individuals than among HIV-monoinfected individuals (Table 2). AIDS-related mortality was higher among HIV/HBV-coinfected individuals and among HIV/HBV/HCV triple-infected individuals compared with HIV-monoinfected individuals but there was little difference in AIDS-related mortality rates between HIV-monoinfected and HIV/HCV-coinfected individuals (Table 2).

**Table 1:** Baseline characteristics of individuals included in the analysis.^a^

	Baseline coinfection status				
	HIV monoinfected	HIV/HBV coinfected	HIV/HCV coinfected	HIV/HBV/HCV triple infected	
	*N* = 22739 *n* (%)	*N* = 1216 *N* (%)	*N* = 1404 *N* (%)	*N* = 127 *N* (%)	*P* value
Median age (years; IQR)	37 (31–44)	39 (33–44)	39 (34–44)	37 (33–42)	<0.001
Ethnicity					
White	13913 (61.1)	625 (51.4)	1148 (81.8)	99 (78.0)	<0.001
Black African	5001 (22.0)	368 (30.3)	68 (4.8)	11 (8.7)	
Other/unknown	3825 (16.8)	223 (18.3)	188 (13.4)	17 (13.4)	
HIV exposure group					
MSM	14102 (62.0)	723 (59.5)	602 (42.9)	63 (49.6)	<0.001
IDU	172 (24.5)	15 (1.2)	483 (34.4)	33 (26.0)	
Male heterosexual	2607 (11.5)	225 (18.7)	105 (7.5)	16 (12.6)	
Female heterosexual	4484 (19.7)	190 (15.6)	110 (7.8)	9 (7.1)	
Other/unknown	1374 (6.0)	61 (5.0)	104 (7.4)	6 (4.7)	
Median CD4^+^ cell count (cells/μl; IQR)	414 (272–582)	357 (227–540)	388 (243–566)	371 (203–530)	<0.001
HIV viral load (copies/ml)					
<50	8459 (37.2)	504 (41.5)	550 (39.2)	51 (40.2)	0.004
≥50	12407 (54.6)	609 (50.1)	768 (54.7)	67 (50.8)	
Unknown	1873 (8.2)	103 (8.5)	86 (6.1)	9 (7.1)	
cART regimen					
NNRTI based	4757 (20.9)	271 (22.3)	250 (17.8)	22 (17.3)	<0.001
PI based	2409 (10.6)	136 (11.2)	189 (13.5)	18 (14.2)	
Other regimen	2847 (12.5)	235 (19.3)	224 (16.0)	25 (20.0)	
Not on ART	12726 (56.0)	574 (47.2)	741 (52.8)	62 (48.8)	
Year of entry into UK CHIC					
1996–1999	7341 (32.3)	493 (40.5)	615 (43.8)	55 (43.3)	<0.001
2000–2004	6072 (26.7)	309 (25.4)	327 (23.3)	23 (18.1)	
2005–2009	7196 (31.7)	322 (26.5)	378 (26.9)	37 (29.1)	
≥2010	2130 (9.4)	92 (7.6)	84 (6.0)	12 (9.5)	
Died during follow-up					
Yes	816 (3.6)	87 (7.2)	145 (10.3)	17 (13.4)	<0.001
No	21923 (96.4)	1129 (92.9)	1259 (89.7)	110 (86.6)	

^a^IQR, interquartile range; cART, combination antiretroviral therapy; NNRTI, nonnucleoside reverse transcriptase inhibitor; PI, protease inhibitor; UK CHIC, UK collaborative HIV cohort study.

**Table 2:** All-cause, liver-related, and AIDS-related mortality, 2004–2012, by hepatitis infection status.

			All-cause mortality		Liver-related mortality		AIDS-related mortality	
HIV/hepatitis infection	Number of individuals	Person-years follow-up	Number of deaths	Mortality rate^a^ (95% CI)	Number of deaths	Mortality rate^a^ (95% CI)	Number of deaths	Mortality rate^a^ (95% CI)
HIV	22 739	103 057	761	7.4 (6.9–7.9)	27	0.3 (0.2–0.4)	164	1.6 (1.4–1.9)
HIV/HBV	1427	6933	96	13.8 (11.2–16.9)	24	3.5 (2.2–5.2)	15	2.2 (1.2–3.6)
HIV/HCV	2325	10 180	172	16.9 (14.5–19.6)	34	3.3 (2.3–4.7)	13	1.3 (0.7–2.2)
HIV/HBV/HCV	435	1644	36	21.9 (15.3–30.3)	10	6.1 (2.9–11.2)	6	3.6 (1.3–7.9)
Total	25 486	121 814	1065	8.7 (8.2–9.3)	95	0.8 (0.6–1.0)	198	1.6 (1.4–1.9)

CI, confidence interval; HBV, hepatitis B virus; HCV, hepatitis C virus.

^a^Mortality rates per 1000 person-years of follow-up.

### Association of hepatitis coinfection with mortality

All categories of hepatitis coinfection were significantly associated with an increased risk of all-cause mortality both in univariable and multivariable analysis (Table 3). In multivariable analysis the greatest increase in risk of mortality was seen among individuals who were HIV/HBV/HCV triple infected. These individuals had a risk of dying 2.29 times as high as that among HIV-monoinfected individuals (95% CI 1.62–3.24). The risk of dying among HIV/HBV and HIV/HCV-coinfected individuals was 1.60 (95% CI 1.27–1.99) and 1.42 (1.15–1.76) times as high as that among HIV-monoinfected individuals, respectively.

**Table 3:** Results from adjusted and unadjusted Poisson regression assess the association of HIV/hepatitis co with all-cause, liver-related, and AIDS-related mortality.

		Unadjusted		Adjusted^a^	
Outcome	Hepatitis coinfection	Risk ratio (95% CI)	*P* value	Risk ratio (95% CI)	*P* value
All-cause mortality	HIV monoinfected	1	–	1	–
	HIV/HBV coinfected	1.88 (1.52–2.32)	<0.0001	1.60 (1.28–2.00)	<0.0001
	HIV/HCV coinfected	2.29 (1.94–2.70)	<0.0001	1.42 (1.15–1.76)	<0.0001
	HIV/HBV/HCV triple infected	2.97 (2.12–4.14)	<0.0001	2.29 (1.62–3.24)	<0.0001
Liver-related mortality	HIV monoinfected	1	–	1	–
	HIV/HBV coinfected	13.21 (7.62–22.90)	<0.0001	10.42 (5.78–18.80)	<0.0001
	HIV/HCV coinfected	12.74 (7.69–21.12)	<0.0001	6.20 (3.31–11.60)	<0.0001
	HIV/HBV/HCV triple infected	23.22 (11.24–47.98)	<0.0001	15.19 (6.94–33.24)	<0.0001
AIDS-related mortality	HIV monoinfected	1	–	1	–
	HIV/HBV coinfected	1.36 (0.80–2.30)	0.25	1.07 (0.63–1.83)	0.80
	HIV/HCV coinfected	0.80 (0.46–1.41)	0.44	0.40 (0.20–0.81)	0.01
	HIV/HBV/HCV triple infected	2.29 (1.02–5.18)	0.05	1.55 (0.65–3.70)	0.32

CI, confidence interval; HBV, hepatitis B virus; HCV, hepatitis C virus.

^a^All models were adjusted for age, HIV exposure group, CD4^+^ cell count, HIV viral load and treatment, and year of follow-up.

The effect of hepatitis coinfection on the risk of liver-related mortality was greater than its effect on all-cause mortality (Table 3). After adjusting for confounders, compared with the liver-related mortality rate in HIV-monoinfected individuals, the risk of liver-related mortality was 10.42 (95% CI 5.78–18.80) times as high among individuals with HIV/HBV coinfection, 6.20 (95% CI 3.31–11.60) times as high among individuals with HIV/HCV coinfection, and 15.19 (95% CI 6.94–33.24) times as high among individuals with HIV/HBV/HCV triple infection.

Hepatitis coinfection was not associated with an increased risk of AIDS-related mortality after adjusting for confounding factors (Table 3). Compared with HIV-monoinfected individuals, there was no significant difference in the risk of AIDS-related mortality among HIV/HBV-coinfected individuals [adjusted rate ratios (ARR) 1.07, 95% CI 0.63–1.83] or among HIV/HBV/HCV triple-infected individuals (ARR 1.55, 95% CI 0.65–3.70). However, HIV/HCV-coinfected individuals had a significantly lower risk of AIDS-related mortality than HIV-monoinfected individuals (ARR 0.40, 95% CI 0.19–0.81).

Some other associations were also observed. In all models, having acquired HIV infection through IDU was a strong predictor of mortality. In addition, increasing age, lower CD4^+^ cell counts, and having a high HIV viral load while being on cART were associated with increased mortality (data not shown).

### Sensitivity analyses

When individuals who acquired their HIV through IDU were excluded from the models of all-cause mortality and liver-related mortality, there were no changes to the direction of the associations between hepatitis coinfection and increased mortality. The effect sizes also remained similar (data not shown). When IDU were excluded from the model of AIDS-related mortality, the association between HIV/HCV coinfection and decreased risk of liver-related mortality became nonsignificant (ARR 0.46, 95% CI 0.19–1.13); however, the direction of the effect remained the same.

## Discussion

In the UK CHIC study, among patients under follow-up in an era of effective treatment for HIV infection, coinfection with HBV or HCV was significantly associated with increased all-cause mortality even after adjusting for other known predictors of mortality. The association between liver-related mortality and coinfection was even more striking. In contrast, HIV/HBV coinfection and HIV/HBV/HCV triple infection was not associated with AIDS-related mortality.

There was some evidence of a protective effect of HCV on the risk of AIDS-related mortality. IDU usually acquire HCV very soon after they start injecting drugs and, therefore, are likely to have been infected with HCV for longer than individuals who have acquired their infections through other routes. IDU also have higher rates of other causes of mortality than individuals who do not inject drugs. Therefore, the protective effect of HCV infection with regard to AIDS-related mortality when IDU are included in the model may be because of long-standing HCV infection, or other mortality specifically associated with injecting behaviours, acting as competing risks for AIDS-related mortality. After excluding IDU from the analysis the effect became nonsignificant; however, the effect size remained the same. It is possible that this is an effect of the recent outbreaks of acute HCV among MSM. These men are likely to be well engaged with HIV care and are therefore less likely to experience AIDS events.

The association of HIV/HBV coinfection with increased all-cause mortality and the even larger effect of HBV infection on the risk of liver-related mortality has been seen in other cohorts [12,14,20,27–29]. This analysis indicates that HIV/HBV-coinfected individuals are 1.6 times more likely to die than HIV-monoinfected individuals. In the largest previously published study comparing mortality among HIV/HBV coinfected and HIV-monoinfected individuals, individuals from the EuroSida cohort who were HIV/HBV coinfected were 1.54 times as likely to die than HIV-monoinfected individuals [27]. The magnitude of increased risk of liver-related mortality among HIV/HBV-coinfected individuals compared with HIV-monoinfected individuals in this UK cohort (10.4 times higher) is greater than previously reported. For example, in the EuroSida study the risk of liver-related mortality among HIV/HBV-coinfected individuals was 3.3 times as high as that among HIV-monoinfected individuals [27]. Similarly, in another large collaborative study (D:A:D) the risk of liver-related mortality was 3.7 times as high in HIV/HBV coinfected compared with HIV-monoinfected individuals. The difference in effect size between the present and previous studies may be because of different levels of liver-related mortality in the monoinfected individuals in each cohort. As UK CHIC has lower proportions of IDU than other cohorts, the rate of liver deaths in the monoinfected group is likely to be lower within UK CHIC, and therefore the additional risk of liver-related mortality among the coinfected group will be greater. Finally, the finding that HIV/HBV coinfection is strongly associated with liver-related mortality but not with AIDS-related mortality is in line with research which has shown that, in the cART era, HBV coinfection is not associated with an increased progression of HIV to AIDS [14,19,27].

The association between increased mortality and HIV/HCV coinfection has also been shown in other cohorts [29–39]. In the present analysis, the risk of all-cause mortality was 1.4 times as high among HIV/HCV-coinfected individuals than that in HIV-monoinfected individuals. This is similar to the effect size seen in a meta-analysis of 20 studies comparing mortality in HIV/HCV coinfected to that in HIV-monoinfected individuals in which HIV/HCV-coinfected individuals had a risk of mortality which was 1.35 times as high as that in HIV-monoinfected individuals [13]. The current study showed a 6.2 times increased risk of liver-related death for HIV/HCV-coinfected individuals compared with HIV-monoinfected individuals. This magnitude of increased risk was smaller than that seen in other cohorts. For example, in the EuroSida cohort the risk of liver-related mortality was 11.7 times higher among HIV/HCV-coinfected individuals than among HIV-monoinfected individuals. This is possibly as a result of the higher proportion of IDU in other cohorts compared with in UK CHIC. As with HIV/HBV coinfection, HIV/HCV coinfection was not associated with AIDS-related mortality. This supports the hypothesis that HIV/HCV coinfection does not alter progression of HIV to AIDS in the era of HAART [37,40].

In assessing the association of hepatitis coinfection with mortality, some other associations were also observed. As expected, IDU and older age and lower CD4^+^ cell count were all significantly associated with increased all-cause, liver-related, and AIDS-related mortality. The observed association between all-cause, liver-related, and AIDS-related mortality and cART/viral load is less intuitive. Being on cART and having a high HIV viral load may be a sign of treatment failure. Alternatively, these could be individuals who have become very unwell from liver disease and have, therefore, opted to stop taking cART. In each of these cases, individuals could be expected to very unwell compared with individuals in the other HIV treatment/viral load categories which might explain the increased risk of mortality.

The study has some limitations. First, although the linkages of the UK CHIC dataset and data from ONS and PHE have resulted in very reliable data on the number of individuals who have died, accurately establishing cause of death is difficult. Therefore, it is possible that the number of AIDS-related and liver-related deaths are underestimated as cause of death data remains incomplete. In addition, data within UK CHIC are obtained from HIV clinics which may be more likely to have a record of a death related directly to HIV (including AIDS-related death) than death from other causes (including liver-related death). However, it is hoped that the use of three sources of death data (UK CHIC, coding causing of death in HIV forms, and PHE/ONS data) have limited introduction of any bias because of differential reporting of deaths within HIV clinics.

Conversely, it is also possible that the number of liver-related deaths is overestimated in coinfected individuals as a known hepatitis diagnosis may influence how a death is reported. In this analysis, the impact of this was minimized by not coding a death as liver related purely on the basis of a mention of viral hepatitis infection and by coding the deaths without knowledge of the individual's hepatitis status.

Efforts to improve the reporting of deaths in UK CHIC have intensified in more recent years. In particular, linkage to PHE data were first conducted using a dataset that contained data up to the end of 2010. Therefore, it is likely that from 2010 onwards a higher proportion of individuals who had died would have information recorded in UK CHIC. This may have introduced some bias with increased deaths having been reported in more recent years.

Although every effort has been made in the analyses presented to adjust for potential confounding factors, there may be some residual confounding. Information on drug use was only available if IDU was the individual's probable route of HIV exposure. Drug use (including non-IDU) is associated with hepatitis coinfection [41,42] and may also increase the risk of death. Similarly, there is no data within UK CHIC on alcohol use. Alcohol use may be higher among coinfected groups than others and is also associated with an increased risk of liver disease and liver-related mortality. Biochemical markers of cirrhosis were not included in the analysis are we believe cirrhosis to be on the causal pathway from hepatitis infection to mortality.

Hepatitis D virus (HDV) is associated with an increased risk of mortality among HIV/HBV-coinfected individuals [43]. The HDV status of HIV/HBV-coinfected individuals was unknown, therefore, we were not able to assess the impact of HDV on mortality among this cohort and the result presented may not represent the effect of HBV infection alone on mortality among HIV-positive individuals. Data on HCV RNA within the dataset were limited and therefore it was not possible to assess the effect of clearance of HCV on mortality in this analysis or to limit the analysis to individuals who have active HCV infection. Similarly, it was not possible to assess the effect of treatment with tenofovir on mortality among HBV-coinfected individuals. Median follow-up for individuals in this study was less than 5 years. This could be considered as a relatively short follow-up when assessing mortality, despite this an association between coinfection and mortality was still observed. Finally, using the data from UK CHIC it is not possible to determine the date of infection with HBV or HCV as individuals may have been infected for some time before their first positive result in the dataset. Therefore, it is not possible to examine the effect of timing of hepatitis infection compared to HIV infection on the clinical outcomes.

Despite these limitations, the analyses presented utilized a large cohort of HIV-positive individuals and, as such, provide estimates of mortality among HIV and hepatitis-coinfected individuals in the United Kingdom. Directly acting agents for treatment of HCV infection are now available which have high rates of cure. If patients are able to access these treatments the increased risk of mortality from HCV infection may be mitigated. The findings of this study highlight the need for primary prevention strategies and the importance of ensuring prompt diagnosis of hepatitis coinfection among HIV-positive individuals and effective linking of coinfected individuals to treatment and monitoring to limit the progression of liver disease.

## Acknowledgements

UK CHIC Steering Committee: Jonathan Ainsworth, Sris Allan, Jane Anderson, Abdel Babiker, David Chadwick, Valerie Delpech, David Dunn, Martin Fisher, Brian Gazzard, Richard Gilson, Mark Gompels, Phillip Hay, Teresa Hill, Margaret Johnson, Sophie Jose, Stephen Kegg, Clifford Leen, Fabiola Martin, Mark Nelson, Chloe Orkin, Adrian Palfreeman, Andrew Phillips, Deenan Pillay, Frank Post, Jillian Pritchard, Caroline Sabin, Achim Schwenk, Anjum Tariq, Roy Trevelion, John Walsh.

UK CHIC Central Co-ordination: University College London (Teresa Hill, Sophie Jose, Andrew Phillips, Caroline Sabin); Medical Research Council Clinical Trials Unit at UCL (MRC CTU at UCL), London (David Dunn, Adam Glabay).

UK CHIC Participating Centres: Brighton and Sussex University Hospitals NHS Trust (M. Fisher, N. Perry, S. Tilbury, E. Youssef, D. Churchill); Chelsea and Westminster Hospital NHS Foundation Trust, London (B. Gazzard, M. Nelson, R. Everett, D. Asboe, S. Mandalia); King's College Hospital NHS Foundation Trust, London (F. Post, H. Korat, C. Taylor, Z. Gleisner, F. Ibrahim, L. Campbell); Mortimer Market Centre, University College London (R. Gilson, N. Brima, I. Williams); Royal Free NHS Foundation Trust/University College London (M. Johnson, M. Youle, F. Lampe, C. Smith, R. Tsintas, C. Chaloner, S. Hutchinson, C. Sabin, A. Phillips, T. Hill, S. Jose, A. Thornton, S. Huntington); Imperial College Healthcare NHS Trust, London (J. Walsh, N. Mackie, A. Winston, J. Weber, F. Ramzan, M. Carder); North Middlesex University Hospital NHS Trust, London (J. Ainsworth, A. Schwenk, S. Miller, C. Wood); The Lothian University Hospitals NHS Trust, Edinburgh (C. Leen, A. Wilson, S. Morris); North Bristol NHS Trust (M. Gompels, S. Allan); Middlesbrough, South Tees Hospitals NHS Foundation Trust (D. Chadwick, E. Cope, J. Gibson); Woolwich, Lewisham and Greenwich NHS Trust (S. Kegg, P. Main, Dr Mitchell, Dr Hunter); Public Health England, London (V. Delpech); UK Community Advisory Board (R. Trevelion).

UK CHIC is funded by the Medical Research Council Grant Numbers G000019, G0600337 G0900274. Additional funding was received from Bristol Myers Squibb, Abbott, Boehringer Ingelheim, Gilead Sciences, and Merck Sharp & Dohme for collection of data regarding coinfected individuals.

Contributions: Study design: A.T., C.A.S.; data analysis: A.T.; first draft of the manuscript: A.T. C.A.S. All authors contributed to the data interpretation, final version of the manuscript and approved the submission.

### Conflicts of interest

There are no conflicts of interest.
